# Diagnostic Yield of the New Bárány Society Criteria for Pediatric Episodic Vestibular Syndrome

**DOI:** 10.3390/jcm14175971

**Published:** 2025-08-23

**Authors:** Mar Rey-Berenguel, Javier Vallecillo-Zorrilla, Edith Karelly Burgueño-Uriarte, María del Carmen Olvera-Porcel, Juan Manuel Espinosa-Sanchez

**Affiliations:** 1 Department of Otolaryngology, Hospital Universitario Virgen de las Nieves, 18014 Granada, Spain; marreyber@correo.ugr.es (M.R.-B.); javivallecillo@correo.ugr.es (J.V.-Z.); kareburgueno@correo.ugr.es (E.K.B.-U.); 2 Biostatistics, Unidad de Gestión y Apoyo a la Investigación, Hospital Universitario Virgen de las Nieves, 18014 Granada, Spain; mariac.olvera.sspa@juntadeandalucia.es; 3 Division of Otolaryngology, Department of Surgery, University of Granada, 18071 Granada, Spain; 4 Otology and Neurotology Group CTS495, Instituto de Investigación Biosanitaria ibs.GRANADA, 18012 Granada, Spain; 5 Sensorineural Pathology Program, Centro de Investigación Biomédica en Red en Enfermedades Raras (CIBERER), 28029 Madrid, Spain

**Keywords:** vestibular diseases, vertigo, migraine disorders, child, adolescent, diagnostic criteria

## Abstract

**Background/Objectives**: Pediatric episodic vestibular syndrome (EVS) is increasingly recognized, with recurrent vertigo of childhood (RVC) and vestibular migraine of childhood (VMC) being the most prevalent disorders. In 2021, the Bárány Society and the International Headache Society proposed new diagnostic criteria for RVC, VMC, and probable VMC (pVMC), replacing the older term benign paroxysmal vertigo (BPV). This study aimed to evaluate the clinical applicability of these new criteria. **Methods**: We conducted a cross-sectional study at a pediatric neurotology clinic within a tertiary hospital, including patients under 18 years with episodic vestibular symptoms evaluated between 2018 and 2025. All patients underwent a standardized neuro-otological assessment. Diagnoses were assigned using both the 2018 ICHD-3 and the 2021 Bárány criteria. Patients who did not fulfill any of the three new diagnostic categories, nor met criteria for any other specific vestibular disorder, were grouped into an undetermined category referred to as episodic vestibular syndrome without hearing loss (EVSw/oHL). Demographic and clinical variables were compared across diagnostic groups using non-parametric and chi-squared tests. **Results**: Among the 202 children evaluated, 109 met the inclusion criteria and were classified as RVC (n = 55), VMC (n = 23), pVMC (n = 13), or EVSw/oHL (n = 18). All patients previously diagnosed with BPV met the new criteria for RVC. Application of the Bárány criteria significantly reduced the proportion of unclassified EVS cases (from 35.78% to 16.51%). Significant clinical differences were observed among the groups in terms of episode duration, presence of vomiting, migraine and headache, and family history of migraine. **Conclusions**: The new Bárány criteria provide a more inclusive and clinically meaningful framework for classifying pediatric EVS. They improve diagnostic clarity, reduce the proportion of unclassifiable cases, and support earlier and more tailored management strategies.

## 1. Introduction

Vestibular symptoms are more common during childhood than previously thought. Population-based studies on children aged 3–17 years reported a 5.3–5.6% prevalence of dizziness and imbalance [1,2]. Although the etiology is varied, recurrent vertigo of childhood (RVC) and vestibular migraine of childhood (VMC) are the most frequent causes of vestibular symptoms in children and adolescents [3,4,5,6,7]. These two disorders were jointly proposed by the Committee for the Classification of Vestibular Disorders of the Bárány Society and the Migraine Classification subgroup of the International Headache Society (IHS) in 2021 [8] (Table 1). According to this proposal, the term RVC replaced the term benign paroxysmal vertigo (BPV) established previously by the IHS in the third edition of the International Classification of Headache Disorders (ICHD-3) [9]. BPV was included in the ICHD-3 among episodic syndromes that may be associated with migraine, formerly known as childhood periodic syndromes. Equally, ICHD-3 also included vestibular migraine (VM) among these episodic syndromes that may be associated with migraine, indicating that it does not involve any age limit. Therefore, the Bárány Society and the IHS proposed the term VMC by simply adding an age criterion to the existing definition. Additionally, the consensus document proposed diagnostic criteria for probable VMC (pVMC) (Table 1). Since the diagnostic criteria for RVC, VMC, and pVMC have been jointly established by the Bárány Society and the IHS, it is expected that these three new disorders will appear in the next edition of the ICHD.

To facilitate clinical interpretation and highlight the conceptual shift introduced by the Bárány Society, a side-by-side comparison of legacy (ICHD-3) and updated criteria is presented in Table 2.

A correct distinction between these disorders is essential for accurate diagnosis and appropriate treatment strategies in pediatric patients with episodic vestibular syndrome (EVS). Therefore, the main objective of this study was to assess whether the diagnostic criteria proposed by the Bárány Society in 2021 improve the classification accuracy of pediatric EVS when compared to the previously established ICHD-3 definitions. We hypothesized that the application of the updated Bárány criteria would reduce the proportion of unclassifiable cases and enable a more precise phenotypic stratification of affected children.

## 2. Materials and Methods

### 2.1. Clinical Study Design and Setting

This observational cross-sectional study was conducted at a tertiary care university hospital between November 2017 and March 2025.

### 2.2. Patient Selection

We evaluated all consecutive patients aged less than 18 years who presented with dizziness, vertigo, or unsteadiness at an outpatient pediatric vertigo clinic. Owing to the COVID-19 pandemic, study enrollment and in-clinic evaluations were paused from March to September 2020.

### 2.3. Clinical Assessment

Patients and their parents were interviewed for a detailed history. All patients with EVS were identified. Subsequently, all patients underwent a comprehensive neuro-otological examination by the senior author, including otoscopy, ocular motility and alignment, ocular convergence, saccades and smooth pursuit, spontaneous nystagmus, gaze-evoked nystagmus, post-rotatory nystagmus, visual vestibular-ocular reflex suppression, positional nystagmus (Dix-Hallpike maneuver, roll test, and center head-hanging position), head-impulse testing, Romberg and Fukuda stepping tests, single-leg stance, past-pointing test, finger-to-nose and finger-to-finger tests, rapid alternating movements, and gait observation. Skull vibration-induced nystagmus was assessed in both children and adolescents, while head-shaking nystagmus was evaluated exclusively in adolescents.

In addition, when deemed necessary, patients were tested for audiovestibular function, including pure-tone audiometry, video head impulse test (vHIT), vestibular evoked myogenic potentials (VEMP), both cervical (cVEMP), and ocular (oVEMP). On the other hand, imaging techniques (brain magnetic resonance imaging or temporal bone computed tomography scan) were performed as needed.

The participants were initially diagnosed according to the ICHD-3 criteria [9]. From 2021 onward, all the new patients were diagnosed according to both the 2018 ICHD-3 criteria and the 2021 diagnostic criteria of the Bárány Society and HIS [8]. Patients seen before 2021 were reclassified according to new criteria after reviewing their medical records. Thus, each subject had two possible diagnoses depending on whether the old or new criteria were applied.

In cases of diagnostic uncertainty, the clinical records were independently reviewed by at least two investigators, and final classification was established by consensus. When disagreement persisted, the senior investigator’s judgment prevailed.

### 2.4. Inclusion Criteria

After thorough evaluation, patients who fulfilled the Bárány Society criteria for VMC, pVMC, and RVC were included in this study. Additionally, we created an undetermined category—episodic vestibular syndrome without hearing loss (EVSw/oHL)—to include patients with episodic vestibular symptoms who failed to meet criteria for RVC, pVMC, or VMC, and whose symptoms could not be attributed to any other vestibular or ICHD-3 diagnosis.

### 2.5. Exclusion Criteria

Patients with other specific diagnoses that may cause EVS —namely, benign paroxysmal positional vertigo (BPPV), episodic ataxia, vestibular paroxysmia, Meniere’s disease, hemodynamic orthostatic dizziness/vertigo, superior canal dehiscence syndrome, perilymphatic fistula, and epilepsy with vestibular aura—were excluded. Patients with migraine with brainstem aura were also excluded.

In all cases assigned to the EVSw/oHL group, these alternative diagnoses were systematically ruled out through an individualized evaluation.

In addition, patients whose legal representatives did not accept or understand their informed consent were excluded.

### 2.6. Data Collection

Demographic and clinical variables were recorded: presenting symptoms, age at symptom onset, age at diagnosis, duration of episodes, presence of headache and migraine, associated hearing loss or other auditory symptoms, presence of vomiting and nausea, and family history of migraine, vertigo, or hearing loss.

The presenting symptoms were recorded in accordance with the Bárány Society definitions [10]. Internal vertigo is the sensation of self-motion when no self-motion is occurring or the sensation of distorted self-motion during an otherwise normal head movement. External vertigo is the false sensation that the visual surround is spinning or flowing. Dizziness is the sensation of disturbed or impaired spatial orientation without a false or distorted sense of motion. Unsteadiness is the feeling of being unstable while seated, standing, or walking without a particular directional preference.

The time intervals used to define episode duration were chosen according to the cutoff values established in the diagnostic criteria for RVC (>1 min) and VMC (>5 min). Additionally, a cut-off of 20 min was included, as this is the minimum duration of Meniere’s disease attacks

### 2.7. Statistical Analysis

A descriptive analysis of the collected variables was performed using measures of central tendency and dispersion for continuous variables, and absolute and relative frequencies for categorical variables. Subsequently, to compare quantitative variables across patient groups, the nonparametric Kruskal–Wallis test was used. Qualitative variables were analyzed using Pearson’s chi-squared test or Fisher’s exact test, as appropriate. Statistical significance was set at *p* < 0.05. When statistical significance was found, pairwise comparisons between groups were conducted with the Bonferroni correction to identify which groups differed.

Calculations were performed using the statistical software STATA version 16.1.

### 2.8. Sample Size and Sampling Procedure

This pilot study was restricted to patients seen in the pediatric and adolescent neurotology clinic at the Hospital Universitario Virgen de las Nieves (Granada, Spain) who presented with dizziness, unsteadiness, or vertigo. Therefore, all patients evaluated at this clinic between November 2017 and March 2025 were included. A consecutive non-probabilistic sampling method was used.

## 3. Results

**Figure 1:**
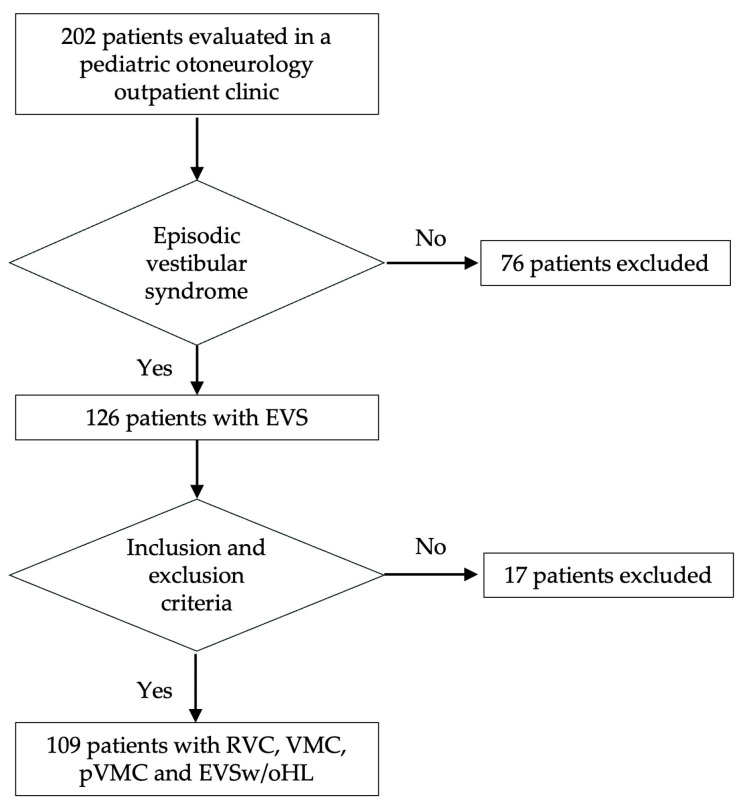
Flowchart representing the patient selection process and diagnostic classification.

We evaluated 202 children and adolescents with dizziness, vertigo, or unsteadiness. The mean age of the patients was 11.164 ± 3.681 years, and 121 (59.9%) were females. We identified 126 patients with pediatric EVS. Finally, 109 subjects were included in the study, with a mean age of 11.47 ± 3.32 years, with 64 (58.72%) females (Figure 1). These patients were classified as having RVC (55, 50.46%), VMC (23, 21.1%), pVMC (13, 11.93%), or EVSw/oHL (18, 16.51%).

**Figure 2:**
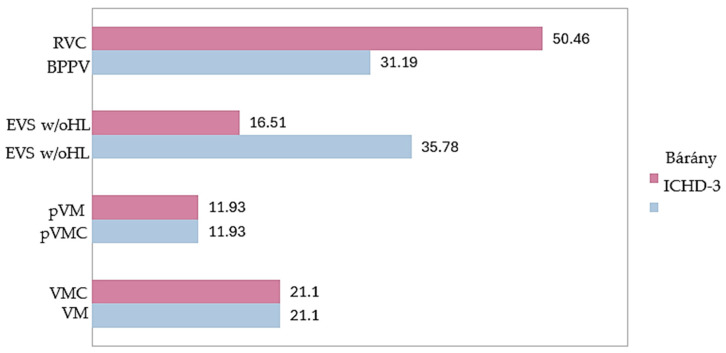
Distribution of patients (%) within each diagnostic category when applying the criteria of the 2018 ICHD-3 with those of the 2021 Bárány Society.

All the patients diagnosed with BPV according to the ICHD-3 criteria met the 2021 Bárány criteria for RVC. All patients diagnosed with VM and pVM were classified as having VMC and pVMC, respectively, according to the new criteria. In addition, 21 (19.27%) children initially diagnosed with EVSw/oHL were re-categorized as having RVC; therefore, the number of patients in the undetermined group was reduced from 35.78% to 16.51% (Figure 2).

The demographic and clinical characteristics of each group are shown in Table 3. Women predominated all diagnostic categories; nevertheless, no statistically significant differences were observed.

We also found no differences in the age of symptom onset; nevertheless, when age at diagnosis was considered, these differences were significant. However, pairwise comparisons between the four categories using the Bonferroni post hoc test did not reveal statistically significant differences in age at diagnosis, although statistical significance was not reached, a non-significant trend was observed towards a difference between the RVC and VMC groups (*p* = 0.069) and between RVC and pVMC (*p* = 0.083).

External vertigo was the most common symptom (75.23%), with no significant differences among the four groups. Nevertheless, vomiting, but not nausea, showed statistically significant differences among the four groups (*p* = 0.002), with differences between the VMC and EVSw/oHL groups (*p* = 0.034), and between the pVMC and EVSw/oHL groups (*p* = 0.004).

Tinnitus was the most frequent auditory symptom, reported in 21.1% of all patients; however, no statistically significant differences were found between diagnostic groups.

Headache was reported by 40% of patients in the RVC group, 100% in the VMC and pVMC groups, and 55.56% in the EVSw/oHL group, with statistically significant differences (*p* = 0.000). When comparing the groups, these differences were significant between the VMC and pVMC groups with respect to the RVC and EVSw/oHL groups.

According to the diagnostic criteria, no patient with RVC reported migraine compared to 100% of the patients with VMC, 69.23% of the patients with pVMC, and 27.78% of those classified as EVSw/oHL; these differences were statistically significant (*p* = 0.000). When comparing between groups, the differences were statistically significant between RVC and VMC (*p* = 0.000), pVMC (*p* = 0.000), and EVSw/oHL (*p* = 0.003), as well as between VMC and EVSw/oHL (*p* = 0.000).

On the other hand, a family history of migraine was reported in 23 (41.82%) children with RVC, 19 (82.61%) with VMC, 9 (69.23%) with pVMC, and 11 (61.11%) with EVSw/oHL, resulting in statistically significant differences (*p* = 0.006). The observed differences between the VMC and RVC groups were statistically significant (*p* = 0.007).

The other variables were not statistically significant.

Table 4 shows the distribution of vestibular episode durations across diagnostic groups. In the RVC group, more than half of the patients (54.55%) experienced episodes between 1 and 5 min, 18.18% reported durations of 5 to 20 min, and 10.91% had episodes lasting 20 to 60 min. Only 16.36% described attacks exceeding one hour. In the VMC group, episode duration was more heterogeneous: 39.13% of patients had episodes longer than 60 min, another 39.13% reported intermediate durations of 5 to 20 min, and 21.74% experienced episodes of 20 to 60 min. In the pVMC group, most patients (76.92%) had episodes of 5 to 20 min, while the remaining 23.08% reported attacks lasting more than one hour. Notably, no patients in this group experienced episodes shorter than five minutes. In contrast, the vast majority of children in the EVSw/oHL group (94.44%) reported very brief episodes lasting less than one minute.

Table 5 presents a descriptive analysis of the EVSw/oHL group, whereas Table A1 provides a detailed breakdown of the characteristics of each of the 18 patients in this group.

## 4. Discussion

Our findings confirm that the updated Bárány Society criteria improve the diagnostic classification of pediatric EVS, particularly by reducing the proportion of unclassifiable cases. Specifically, the application of the new Bárány criteria decreased the proportion of unclassifiable cases (EVSw/oHL) from 35.78% to 16.51%, representing a relative reduction of over 50%. This diagnostic gain was primarily attributable to two key changes: (1) the inclusion of shorter episodes (≥1 min) within the new definition of RVC, and (2) the explicit exclusion from this category of patients meeting criterion B or C for VMC—namely, those with a current or past history of migraine or with vestibular episodes accompanied by migraine features. The previous diagnostic label of BPV lacked a clearly defined minimum duration threshold, referring only to “brief episodes.” Consequently, children with episodes lasting 1 to 4 min could not meet VM criteria because of insufficient duration, while the BPV definition—without a specific lower threshold—provided limited diagnostic clarity for this group. Introducing a one-minute minimum duration in the RVC definition improves diagnostic precision for these patients. In addition, BPV is now fully encompassed within the RVC category, and VM and VMC are considered equivalent entities, differing only in age cut-off. The updated pVMC criteria, which require only three episodes rather than five as in the former pVM and VM definitions, also improve clinical applicability, especially in children evaluated early in the disease course [8].

According to the ICHD-3, BPV is defined as at least five episodes of spontaneous vertigo occurring without warning and resolving within minutes to hours, in the absence of loss of consciousness [9]. Diagnosis also requires at least one accompanying symptom or sign—such as nystagmus, ataxia, vomiting, pallor, or fear—and normal neurological, auditory, and vestibular function between episodes. Notably, the previous requirement for a normal electroencephalogram, included in ICHD-2 under the term benign paroxysmal vertigo of childhood (BPVC), was removed in the current ICHD-3 revision.

RVC is a broader clinical entity that does not require associated symptoms or signs for diagnosis. The main distinction between RVC and VMC lies in the exclusion of both a personal history of migraine (criterion B) and migraine features during vestibular episodes (criterion C), as defined by the Bárány Society. Therefore, a precise understanding of migraine diagnostic criteria—particularly their pediatric manifestations—is essential for accurately distinguishing RVC from VMC in clinical practice.

According to the Bárány Society criteria, episode duration is a key parameter for differentiating RVC from both VMC and pVMC. In VMC and pVMC, vestibular symptoms must last more than five minutes, whereas RVC includes episodes as short as one minute. This change clarifies previous ambiguities surrounding the term BPV, which traditionally referred to vertigo episodes lasting from several minutes to hours, without a clearly defined lower limit. In current clinical usage, however, the term *paroxysmal* is typically reserved for vestibular events lasting less than one minute, such as those seen in BPPV or vestibular paroxysmia. Introducing a one-minute minimum duration in the RVC definition resolves this terminological inconsistency and improves diagnostic clarity for short-duration episodes in children.

Our findings showed clear differences in vestibular episode duration across diagnostic categories. Children with RVC typically experienced shorter episodes, most often lasting between 1 and 5 min. In contrast, those diagnosed with pVMC or VMC reported longer durations, frequently in the 5–20 min range or exceeding one hour. Notably, patients with VMC had the longest episodes, consistent with the findings of Teggi et al. [11], supporting the internal consistency of the Bárány classification.

The current ≥5 min duration threshold required for diagnosing VMC excludes certain clinical cases that otherwise fulfill criterion B (personal history of migraine) and/or criterion C (migraine features during episodes). In our cohort, nearly two-thirds of EVSw/oHL patients (11/18; 61.1%) could not be classified as RVC because their vestibular episodes lasted less than one minute (Table A1). Moreover, five patients in this group experienced migraine headaches during brief episodes lasting only a few seconds—insufficient for VMC or pVMC classification. Interestingly, the Bárány Society acknowledges that some VMC or pVMC attacks may last only seconds, despite the formal threshold of five minutes [8]. This discrepancy highlights a potential limitation in the current duration criterion. Additionally, patient A14 reported unsteadiness, a symptom not included under criterion A for VMC, further illustrating the challenges in classifying borderline cases.

Patient A12 met the minimum duration requirement but reported bilateral tinnitus during each vestibular episode. Although this auditory symptom prompted provisional classification as EVSw/oHL, it is important to note that the presence of tinnitus does not exclude a diagnosis of RVC. This case underscores the value of longitudinal follow-up in resolving diagnostic uncertainty.

Patient A18 was classified as EVSw/oHL because the number of vestibular episodes did not meet the minimum required for a diagnosis of vestibular paroxysmia [12]. Importantly, this patient also failed to meet the criteria for RVC (episodes <1 min), pVMC/VMC (absence of migraine features), or any other ICHD-3/Bárány Society diagnosis, as confirmed by a thorough evaluation including audiogram, brain MRI, and EEG. These individual observations prompted a descriptive analysis of the entire EVSw/oHL group (Table 5).

The descriptive analysis of the EVSw/oHL group (Table 5) showed that almost all patients (94.4%) experienced episodes lasting less than one minute, below the minimum duration threshold required for both RVC and pVMC/VMC according to the Bárány Society classification. Moreover, more than one quarter of patients (27.8%) exhibited migraine features, and over half (61.1%) reported a family history of migraine. This combination of very brief episodes and migraine-related traits—either clinical or familial—may account for the difficulty in classifying these patients as RVC, pVMC, or VMC, despite their recurrent vestibular symptoms. These findings illustrate the diagnostic challenges posed by this subgroup and underscore the need for longitudinal follow-up to determine whether some of these patients eventually meet the criteria for a defined syndrome. They also highlight potential limitations of the current Bárány Society criteria—particularly the lower duration threshold for VMC/pVMC and the exclusion of imbalance or dizziness as qualifying symptoms—which may contribute to the number of unclassifiable cases in pediatric populations.

These cases demonstrate that multiple follow-up visits are often necessary to achieve a definitive diagnosis, particularly as some patients initially classified as pVM may evolve into VMC over time. Given the difficulties many children face in articulating vestibular symptoms, longitudinal clinical assessment remains essential for diagnostic precision.

In our cohort, most patients diagnosed with RVC were female; however, this difference did not reach statistical significance when compared with other diagnostic categories [11,13,14]. Interestingly, Dunker et al. [15] reported that in female patients, RVC episodes tend to have a later onset, occur more frequently, and last longer.

The mean age at symptom onset in the RVC group was 9.15 ± 3.43 years, consistent with previous studies [13,16], although some authors have reported earlier onset [15,17]. This contrasts with Basser’s original description of BPVC, which typically began before age four and rarely after age eight [18]. In our cohort, age at onset was lower in RVC patients than in those with VM (10.91 ± 2.35 years), consistent with earlier reports [4,11,13]; however, this difference was not statistically significant when comparing across all four diagnostic groups. Teggi et al. also noted that in pediatric patients, migraine onset tends to precede vertigo by approximately 18 months—shorter than the interval usually observed in adult VM [11].

Statistically significant differences in age at diagnosis were found across the four diagnostic groups; however, Bonferroni post hoc comparisons did not reach significance. There was a non-significant trend between the RVC and VMC groups (*p* = 0.069) and between RVC and pVMC (*p* = 0.083). These trends may reflect true underlying differences that did not reach statistical significance, potentially due to the limited sample size in each subgroup.

Most patients with EVS (75.23%) in our series reported external vertigo, with no statistically significant differences between diagnostic groups. Specifically, 74.6% of RVC patients experienced vertigo, consistent with previous studies [15]. Interestingly, Teggi et al. [11] reported that none of the RVC patients in their cohort described either external or internal vertigo, underscoring possible discrepancies in symptom reporting or interpretation across studies.

In VMC, only a specific subset of vestibular symptoms qualifies for diagnosis: spontaneous vertigo, positional vertigo, visually induced vertigo, head motion–induced vertigo, and head motion-induced dizziness with nausea [19]. Other complaints—such as positional dizziness, unsteadiness, and motion sensitivity—although frequently reported by VMC patients, are not included in the formal diagnostic criteria. As a result, one of our patients who fulfilled all other VMC criteria was classified as EVSw/oHL because the predominant symptom was unsteadiness (A14), and another (A17) because the dizziness occurred without nausea (Table A1).

In contrast, light-headedness and spinning vertigo have been reported in 53% and 27.7% of children with migraine, respectively [20].

In our cohort, auditory symptoms were reported more often by patients with pVMC, although the differences among diagnostic groups did not reach statistical significance. Similarly, Zhang et al. [14] observed no significant differences, despite a higher frequency of auditory symptoms in their RVC group. In contrast, Teggi et al. [11] found auditory symptoms to be more common in VMC than in pVMC, and absent altogether in patients with RVC.

In our cohort, 40% of patients reported non-migraine headaches. This prevalence was higher than that reported by Dunker et al. [15], who found 26% among their RVC patients during vertigo episodes, and also exceeded the 23% reported in the same study. Balzanelli et al. [21] found a prevalence of 17.5%, closely matching the 17.3% reported by Zhang et al. [14].

Of the 13 patients diagnosed with pVMC, 4 (30.8%) did not meet criterion B (absence of a personal history of migraine), and 9 (69.3%) did not meet criterion C, which requires migraine features during vestibular episodes. Longitudinal follow-up will be essential to determine how many of these patients eventually meet full criteria for VMC.

In our series, a family history of migraine was reported in 23 children with RVC (41.8%), 9 with pVMC (69.2%), and 19 with VMC (82.6%), with statistically significant differences between the VMC and RVC groups (*p* = 0.007). These findings are consistent with those of Teggi et al. [11] and with a systematic review reporting a 75.65% prevalence in VMC [22]. By contrast, both Balzanelli et al. and Dunker et al. reported a 57% prevalence in patients with VMC, while Balzanelli found lower rates—35%—in those with BPVC [15,21]. Notably, Zhang et al. [14] observed much lower rates: 22.2% in VMC, 9.4% in pVMC, and 0% in RVC. The hypothesis that BPV may represent a migraine precursor has also been proposed [23,24,25]. This variability underscores the need for larger, multicenter studies, as both our data and those of others indicate phenotypic overlap between RVC and VMC, despite the mutually exclusive migraine criteria that define these categories.

The unexpectedly high prevalence of a family history of migraine in RVC patients (41.8%) suggests that this category may encompass more than one pathophysiological subtype. A potential subdivision could include a “migraine-related RVC” subtype—characterized by family history and minor migrainous traits falling short of VMC or pVMC criteria—and a “non-migrainous RVC” subtype, in which such features are entirely absent. This distinction might be clinically relevant, as children in the migraine-related RVC group could potentially benefit from anticipatory guidance regarding migraine evolution and closer follow-up.

Our data support the concept of a phenotypic continuum linking RVC, pVMC, and VMC, in which migraine features gradually emerge over time. In this model, RVC would represent the mildest end of the spectrum, VMC the most fully expressed phenotype, and pVMC an intermediate stage [8]. A better understanding of this continuum may enable earlier identification of children at risk for developing vestibular migraine and provide opportunities for more personalized management strategies.

This study did not include detailed neurotological examination findings or vestibular function test results, as the primary objective was to characterize patients based solely on symptomatology in accordance with current consensus criteria. Nonetheless, these evaluations were performed systematically to rule out alternative diagnoses and to confirm that each case was not better explained by another ICHD-3 diagnosis or vestibular disorder. In selected cases, additional imaging studies were performed when clinically indicated.

Children with RVC generally do not exhibit interictal signs or symptoms, although occasional findings such as positional nystagmus and saccadic vertical smooth pursuit have been reported [15,25]. Similarly, neurotological examination in children with VMC is typically normal between episodes, although mild ocular motor abnormalities—such as saccadic smooth pursuit or central positional nystagmus—may sometimes be observed [11]. Although some authors have reported abnormalities on vHIT and caloric testing, no statistically significant differences have been found among the RVC, pVMC, and VMC groups [13,14,26], suggesting that these tests offer limited value for differential diagnosis. Likewise, although VEMP abnormalities appear more frequently in RVC in some series, these findings have not reached statistical significance and therefore cannot be used reliably to distinguish between diagnostic categories [14,27,28].

One of the main limitations of this study is the relatively small sample size within each diagnostic subgroup, which reduces statistical power and may explain the absence of significant differences in certain comparisons. Additionally, the lack of detailed data on the number and frequency of episodes limits our ability to assess and compare symptom burden across groups. It is also important to acknowledge the inherent difficulty younger children may have in accurately describing their symptoms and estimating episode duration.

Future multicenter studies with larger patient cohorts will not only increase statistical power but also allow for more sophisticated subgroup analyses—such as cluster analysis—to investigate the phenotypic heterogeneity within the RVC group. Another limitation is the cross-sectional design, which does not allow assessment of diagnostic stability or progression between categories (e.g., pVMC evolving into VMC). Addressing this question would require prospective longitudinal studies specifically designed for follow-up, which should be prioritized in future research.

Although our study was not designed to assess the clinical consequences of diagnostic reclassification, the application of the Bárány Society criteria enabled more precise diagnostic labeling. This improved classification facilitated more targeted patient counseling and individualized follow-up planning. Future prospective studies should examine whether such diagnostic refinement results in changes to therapeutic decision-making, improved symptom control, or better quality of life.

## 5. Conclusions

There are currently no biomarkers for RVC or VMC; thus, diagnosis remains clinical, relying on symptoms reported by the child, parents, or caregivers, and on the exclusion of other conditions through appropriate investigations. A comprehensive medical history and physical examination, complemented when necessary by audiovestibular testing and/or neuroimaging, are essential for the accurate diagnosis and management of pediatric EVS.

The new diagnostic criteria proposed by the Bárány Society improve diagnostic accuracy and significantly reduce the proportion of unclassified pediatric EVS cases. By providing clearer definitions of episode duration and incorporating migraine-related features, these criteria enable better patient stratification and may enhance both clinical decision-making and longitudinal follow-up strategies.

## Figures and Tables

**Table 1:** Diagnostic criteria for pediatric episodic vestibular syndromes according to Bárány Society and ICHD-3.

Clinical Entity	Diagnostic Criteria
RVC ^1^	A. At least three episodes with vestibular symptoms of moderate or severe intensity, lasting between 1 min and 72 h B. None of the criteria B and C for vestibular migraine of childhood C. Age < 18 years D. Not better accounted for by another headache disorder, vestibular disorder, or other condition
VMC ^2^	A. At least five episodes with vestibular symptoms of moderate or severe intensity, lasting between five minutes and 72 h B. A current or past history of migraine with or without aura C. At least half of episodes are associated with at least one of the following three migraine features: 1. Headache with at least two of the following four characteristics: (a) One-sided location (b) Pulsating quality (c) Moderate or severe pain intensity (d) Aggravation by routine physical activity 2. Photophobia and phonophobia 3. Visual aura D. Age < 18 years E. Not better accounted for by another headache disorder, vestibular disorder, or other condition
pVMC ^3^	A. At least three episodes with vestibular symptoms of moderate or severe intensity, lasting between five minutes and 72 h B. Only one of the criteria B and C for vestibular migraine of childhood C. Age < 18 years D. Not better accounted for by another headache disorder, vestibular disorder, or other condition
BPV ^4^	A. At least five attacks fulfilling criteria B and C B. Vertigo occurring without warning, maximal at onset, and resolving spontaneously after minutes to hours without loss of consciousness C. At least one of the following five associated symptoms or signs: 1. nystagmus 2. ataxia 3. vomiting 4. pallor 5. fearfulness D. Normal neurological examination and audiometric and vestibular functions between attacks E. Not attributed to another disorder.
VM ^5^	A. At least 5 episodes with vestibular symptoms of moderate or severe intensity, lasting 5 min to 72 h B. Current or previous history of migraine with or without aura according to the International Classification of Headache Disorders (ICHD) C. One or more migraine features with at least 50% of the vestibular episodes: 1. headache with at least two of the following characteristics: (a) one-sided location (b) pulsating quality, (c) moderate or severe pain intensity (d) aggravation by routine physical activity 2. photophobia and phonophobia 3. visual aura D. Not better accounted for by another vestibular or ICHD diagnosis
pVM ^6^	A. At least 5 episodes with vestibular symptoms of moderate or severe intensity, lasting 5 min to 72 h. B. Only one of the criteria B and C for vestibular migraine is fulfilled (migraine history or migraine features during the episode). C. Not better accounted for by another vestibular or ICHD diagnosis.

^1^ RVC: Recurrent vertigo of childhood; ^2^ VMC: Vestibular migraine of childhood; ^3^ pVMC: Probable vestibular migraine of childhood; ^4^ BPV: Benign paroxysmal vertigo; ^5^ VM: Vestibular migraine; ^6^ pVM: Probable vestibular migraine. RVC, VMC, and pVMC according to the Bárány Society (2021); BPV and VM following ICHD-3 (2018); pVM as defined by the Bárány Society in 2012.

**Table 2:** Comparison between legacy (ICHD-3, 2018) and updated (Bárány Society, 2021) diagnostic criteria for pediatric episodic vestibular syndromes. The Bárány criteria introduce explicit lower duration thresholds, stricter migraine-related criteria, and an age restriction (<18 years) that distinguish RVC, pVMC, and VMC as mutually exclusive entities. BPV has been replaced by RVC.

Diagnostic Criterion	ICHD-3, 2018	Bárány Society, 2021
Minimum number of episodes	≥5 (BPV and VM)	RVC and pVMC: ≥3 episodes VMC: ≥5 episodes
Duration of episodes	BPV: minutes to hours (no lower limit) VM: 5 min to 72 h	RVC: ≥1 min pVMC and VMC: ≥5 min to 72 h
Migraine history	VM: required BPV: not defined	VMC: required RVC: absent
Migraine features during episodes	VM: required (≥50% episodes)	VMC: required RVC: absent
Allowed symptoms	BPV: vertigo ± signs/other symptoms (nystagmus, ataxia, vomiting, pallor, fear) VM: specified vestibular symptoms	RVC: vestibular symptoms VMC, pVMC: specified vestibular symptoms
Age restrictions	BPV: <18 y VM: any age	All three (RVC, pVMC, VMC) apply <18 y
Terminology	BPV (childhood) pVMC and VM (all ages)	BPV replaced by RVC VMC and pVMC

**Table 3:** Demographics and clinical characteristics by group.

	RVC ^1^ N = 55	VMC ^2^ N = 23	pVMC ^3^ N = 13	EVSw/oHL ^4^ N = 18	*p*-Value	Post hoc Test ^∆^
**Women** CI 95%	30 (54.55) 40.554–68.030	14 (60.87) 38.542–80.292	10 (76.92) 46.187–94.962	10 (55.56) 30.757–78.470	0.528	
**Onset** *(yrs)* ** CI 95%	10 (5) 8.201–10.090	10 (3) 9.895–11.931	12 (4) 9.037–12.809	10.5 (3) 8.151–11.515	0.131	
**Diagnosis** *(yrs)* ** CI 95%	11 (4) 8.939–10.843	12 (4) 10.992–12.834	13 (3) 9.977–14.177	11 (4) 8.913–12.309	0.044	1 vs. 2: *p* = 0.069 1 vs. 3: *p* = 0.083
External vertigo * CI 95%	41 (74.55) 60.997–85.330	16 (69.57) 47.080–86.790	12 (92.31) 63.970–99.805	13 (72.22) 46.520–90.305	0.479	
Internal vertigo * CI 95%	4 (7.27) 2.017–17.587	3 (13.04) 2.775–33.589	2 (15.38) 1.921–45.447	0 (0)	0.292	
Dizziness * CI 95%	11 (20) 10.430–32.973	7 (30.43) 13.210–52.919	1 (7.69) 0.195–36.030	5 (27.78) 9.694–53.480	0.397	
Unsteadiness * CI 95%	19 (34.55) 22.237–48.581	13 (56.52) 34.494–76.809	3 (23.08) 5.038–53.813	16 (33.33) 13.343–59.007	0.188	
Posit. vertigo * CI 95%	3 (5.45) 1.139–15.124	1 (4.35) 0.110–21.949	2 (15.38) 1920–45.447	3 (16.67) 3.579–41.418	0.243	
Visual vertigo * CI 95%	1 (1.82) 0.046–9.719	0 (0)	2 (15.38) 1.921–45.447	1 (5.56) 0.141–27.294	0.082	
Hearing loss * CI 95%	2 (3.64) 0.443–12.526	0 (0)	1 (7.69) 0.019–36.030	1 (5.56) 0.141–27.294	0.549	
Tinnitus * CI 95%	11 (20) 10.430–32.973	5 (21.74) 7.460–43.703	4 (30.77) 9.092–61.426	3 (16.67) 3.579–41.418	0.796	
Ear fullness * CI 95%	3 (5.45) 1.140–15.124	3 (13.04) 2.775–33.589	1 (7.69) 0.194–36.030	0 (0)	0.374	
Headache * CI 95%	22 (40) 27.023–54.093	23 (100) 85.181–100	13 (100) 75.295–100	10 (55.56) 30.747–78.470	0.000	2 vs. 4: *p* = 0.003 2 vs. 1: *p* = 0.000 3 vs. 1: *p* = 0.000 3 vs. 4: *p* = 0.058
Migraine * CI 95%	0 (0)	23 (100) 85.181–100	9 (69.23) 38.574–90.908	5 (27.78) 9.695–53.480	0.000	2 vs. 4: *p* = 0.000 2 vs. 1: *p* = 0.000 3 vs. 1: *p* = 0.000 4 vs. 1: *p* = 0.003
Nausea * CI 95%	21 (38.18) 25.409–52.273	10 (43.48) 23.191–65.505	9 (69.23) 38.574–90.908	4 (22.22) 6.409–47.637	0.072	
Vomiting * CI 95%	13 (23.64) 13.228–37.020	8 (34.78) 16.376–57.266	7 (53.85) 25.135–80.777	0 (0)	0.002	2 vs. 4: *p* = 0.034 3 vs. 4: *p* = 0.004
FH migraine * CI 95%	23 (41.82) 28.654–55.894	19 (82.61) 61.122–95.049	9 (69.23) 38.574–90.908	11 (61.11) 35.745–82.701	0.006	2 vs. 1: *p* = 0.007
FH HL * CI 95%	10 (18.18) 9.080–30.905	3 (13.04) 2.775–33.589	0 (0)	3 (16.67) 3.579–41.418	0.457	
FH vertigo * CI 95%	22 (40) 27.023–54.093	6 (26.09) 10.229–48.405	2 (15.38) 1.921–45.447	2 (11.11) 1.375–34.712	0.075	

^1^ RVC: recurrent vertigo of childhood; ^2^ VMC; vestibular migraine of childhood; ^3^ pVMC: probable vestibular migraine of childhood; ^4^ EVSw/oHL4: episodic vestibular syndrome without hearing loss * Qualitative variables: n (%); χ^2^ or Fisher test ** Quantitative variables: median (interquartile range); Kruskal-Wallis test. **^∆^** Using the Bonferroni post hoc comparison test. Onset: age at onset; diagnosis: age at diagnosis; posit. vertigo: positional vertigo; FH migraine: family history of migraine; FH HL: family history of hearing loss; FH vertigo: family history of vertigo; CI: 95% confidence interval.

**Table 4:** Duration of vestibular episodes among the different groups.

	RVC ^1^ N = 55	VMC ^2^ N = 23	pVMC ^3^ N = 13	EVSw/oHL ^4^ N = 18
<1 min * CI 95%	0 (0)	0 (0)	0 (0)	17 (94.44) 72.706–99.860
1–5 min * CI 95%	30 (54.55) 40.554–68.030	0 (0)	0 (0)	0 (0)
5–20 min * CI 95%	10 (18.18) 9.070–30.905	9 (39.13) 19.708–61.458	10 (76.92) 46.187–94.962	1 (5.56) 0.140–27.294
20–60 min * CI 95%	6 (10.91) 4.110–22.471	5 (21.74) 7.460–43.703	0 (0)	0 (0)
>60 min * CI 95%	9 (16.36) 7.767–28.803	9 (39.13) 19.708–61.458	3 (23.08) 5.038–53.813	0 (0)

^1^ RVC: recurrent vertigo of childhood; ^2^ VMC; vestibular migraine of childhood; ^3^ pVMC: probable vestibular migraine of childhood; ^4^: EVSw/oHL: episodic vestibular syndrome without hearing loss; CI: 95% confidence interval. * Qualitative variables: n (%).

**Table 5:** Descriptive analysis of the episodic vestibular syndrome without hearing loss group (EVSw/oHL).

	EVSw/oHL ^1^ N = 18
Nº episodes *	24.28 (19)
Dizziness **	1 (5.56)
Unsteadiness **	1 (5.56)
Vertigo **	16 (88.89)
Duration of episodes **	
<1 min	17 (94.44)
5–20 min	1 (5.56)
Plausible diagnosis **	
RVC	12 (66.67)
VMC	5 (27.78)
Vestibular paroxysmia	1 (5.56)
Auditory symptoms **	3 (16.67)
Migraine **	5 (27.78)

^1^ EVSw/oHL: episodic vestibular syndrome without hearing loss. RVC: recurrent vertigo of childhood; VMC: vestibular migraine of childhood. * Quantitative variables: median (interquartile range); ** Qualitative variables: n (%).

## Data Availability

De-identified data are available on request, preceded by a signed data access agreement form.

## Abbreviations

The following abbreviations are used in this manuscript:

BPPV	Benign paroxysmal positional vertigo
BPV	Benign paroxysmal vertigo
BPVC	Benign paroxismal vertigo of childhood
cVEMP	Cervical vestibular evoked myogenic potentials
EEG	Electroencephalogram
EVS	Episodic vestibular syndrome
EVSw/oHL	Episodic vestibular syndrome without hearing loss
ICHD-3	International Classification of Headache Disorders
HIS	International Headache Society
MRI	Magnetic resonance imaging
oVEMP	Ocular vestibular evoked myogenic potentials
pVM	Probable vestibular migraine
pVMC	Probable vestibular migraine of childhood
RVC	Recurrent vertigo of childhood
VEMP	Vestibular evoked myogenic potentials
vHIT	Video head impulse test
VM	Vestibular migraine
VMC	Vestibular migraine of childhood

## Appendix A

**Table A1:** Patients in the vestibular episodic syndrome without hearing loss group (EVSw/oHL).

	Vestibular Symptom	Duration of Episodes	Auditory Symptoms	Migraine	Number of Episodes	Plausible Diagnosis
A1	Vertigo	<1 min	No	No	5	RVC
A2	Vertigo	<1 min	No	No	10	RVC
A3	Vertigo	<1 min	No	No	5	RVC
A4	Vertigo	<1 min	No	No	5	RVC
A5	Vertigo	<1 min	No	No	90	RVC
A6	Vertigo	<1 min	No	No	7	RVC
A7	Vertigo	<1 min	No	No	9	RVC
A8	Vertigo	<1 min	No	No	100	RVC
A9	Vertigo	<1min	No	No	8	RVC
A10	Vertigo	<1 min	No	No	6	RVC
A11	Vertigo	<1 min	Yes	No	8	RVC
A12	Vertigo	5–20 min	Yes	No	4	RVC
A13	Vertigo	<1 min	No	Yes	12	VMC
A14	Unsteadiness	<1 min	No	Yes	24	VMC
A15	Vertigo	<1 min	Yes	Yes	90	VMC
A16	Vertigo	<1 min	No	Yes	40	VMC
A17	Dizziness	<1 min	No	Yes	10	VMC
A18	Vertigo	<1 min	No	No	4	Vestibular paroxysmia

RVC: recurrent vertigo of childhood; VMC: vestibular migraine of childhood; EVSw/oHL: episodic vestibular syndrome without hearing loss.
